# Dynamics of floret initiation/death determining spike fertility in wheat as affected by *Ppd* genes under field conditions

**DOI:** 10.1093/jxb/ery105

**Published:** 2018-03-19

**Authors:** Paula Prieto, Helga Ochagavía, Roxana Savin, Simon Griffiths, Gustavo A Slafer

**Affiliations:** 1 Department of Crop and Forest Sciences and AGROTECNIO (Center for Research in Agrotechnology), University of Lleida, Lleida, Spain; 2 John Innes Centre, Norwich Research Park, Colney Lane, Norwich, UK; 3 ICREA, Catalonian Institution for Research and Advanced Studies, Spain

**Keywords:** Floret development, floret survival, late reproductive phase, photoperiod insensitivity, stem elongation, *Triticum aestivum*

## Abstract

As wheat yield is linearly related to grain number, understanding the physiological determinants of the number of fertile florets based on floret development dynamics due to the role of the particular genes is relevant. The effects of photoperiod genes on dynamics of floret development are largely ignored. Field experiments were carried out to (i) characterize the dynamics of floret primordia initiation and degeneration and (ii) to determine which are the most critical traits of such dynamics in establishing genotypic differences in the number of fertile florets at anthesis in near isogenic lines (NILs) carrying photoperiod-insensitive alleles. Results varied in magnitude between the two growing seasons, but in general introgression of *Ppd-1a* alleles reduced the number of fertile florets. The actual effect was affected not only by the genome and the doses but also by the source of the alleles. Differences in the number of fertile florets were mainly explained by differences in the floret generation/degeneration dynamics, and in most cases associated with floret survival. Manipulating photoperiod insensitivity, unquestionably useful for changing flowering time, may reduce spike fertility but much less than proportionally to the change in duration of development, as the insensitivity alleles did increase the rate of floret development.

## Teaser

*Ppd-1a* reduced the number of fertile florets due largely to lowering floret survival, and the effect was not proportional to the duration of the late reproductive phase.

## Introduction

Gains in the grain yield of wheat can be achieved by improving any of its major components, but significant gains would be almost exclusively dependent on increments in the number of grains per unit land area (Slafer *et al.*, 2014).Grain number is mostly associated with the number of fertile florets produced (Kirby, 1988; Slafer and Andrade, 1993; Miralles *et al.*, 1998, 2000) mainly because wheat is a cleistogamous species. Therefore, grain number would be largely determined by the number of fertile florets which, in turn, is the consequence of floret generation/degeneration processes. The number of fertile florets was reported to be affected by (i) genotypic differences (Miralles *et al.*, 1998) and (ii) environmental conditions such as nutrient availability (Sibony and Pinthus, 1988; Ferrante *et al.*, 2010) and red/far-red light ratios (Ugarte *et al.*, 2010), but mainly photoperiod (Miralles *et al.*, 2000; González *et al.*, 2003, 2005*a*, *b*; Ghiglione *et al.*, 2008; Serrago *et al.*, 2008). Thus, the importance of these environmental signals for these developmental phases lies in the effect they have on the organs that are being generated at that time and the fact that these organs are the building blocks of grain yield (Slafer *et al.*, 1996; Slafer, 2003). Manipulating developmental processes during the critical period when floret development takes place becomes an important tool to increase spike fertility (Fischer, 2011; Foulkes *et al.*, 2011; García *et al.*, 2011; Slafer *et al.*, 2015). As spike fertility is a critical trait to raise yield in wheat further (e.g. Reynolds *et al.*, 2012, Elía *et al.*, 2016) because it seems reasonably heritable (Mirabella *et al.*, 2016) and responsive to selection (Pedro *et al.*, 2012). For these reasons, it is extremely important that we understand how major genes deployed in wheat breeding do or do not affect the dynamics of floret generation/degeneration and consequently spike fertility.

During the early reproductive phase, all spikelets are initiated (see companion paper; Ochagavía *et al.*, 2018). During the late reproductive phase—from the terminal spikelet to anthesis—floret primordia develop within the spikelets and the outcome of this process determines spike fertility. Unlike leaf and spikelet development, in which all initiated organs prosper and are visible in the adult plant, floret development includes a phase of floret initiation followed by a phase of floret death, and the balance between initiation and death determines the number of fertile florets that may produce grains (Kirby, 1988). Usually, 6–12 floret primordia per spikelet are initiated (Sibony and Pinthus, 1988; Youssefian *et al.*, 1992; Miralles *et al.*, 1998), but most of them degenerate before anthesis. The actual magnitude of this mortality (González *et al.*, 2005*b*; Ghiglione *et al.*, 2008; Guo and Schnurbusch, 2015; Guo *et al.*, 2016), as well as the timing of the onset of floret mortality (González *et al.*, 2011; Ferrante *et al.*, 2013), seems likely to be associated with the availability of assimilates to the growing juvenile spikes.

The rate of development of the late reproductive phase, determining the duration of the phase of stem elongation, was reported to be accelerated by extending the photoperiod (González *et al.*, 2003, 2005*a*), which resulted in reductions in spike fertility (Miralles *et al.*, 2000; González *et al.*, 2003, 2005*b*). The corollary of these studies would be that the introgression of photoperiod insensitivity alleles (a resource commonly deployed in breeding programmes to improve adaptation) could then produce a trade-off in spike fertility. However, due to the intrinsic difficulties associated with measurements required, the effects of major *Ppd* alleles on the specific length of the floret development phase immediately preceding flowering have received little attention, and those on the rates and dynamics of floret development have only been exceptionally analysed. In addition to the limited number of studies quantifying the effects of *Ppd* alleles on the physiological bases of floret fertility, a further limitation is the fact that they have considered a very restricted pool of near isogenic lines (e.g. González *et al.*, 2005*c*). As the genetic background may carry developmental response effects (Jones *et al.*, 2016), it is critical to determine the effects of these alleles on the developmental processes determining floret fertility with more comprehensive studies.

We aimed to quantify the effects of *Ppd* alleles on the dynamics of floret developmental patterns to shed light on some physiological causes of the effects of these alleles on the reproductive fertility of the crop. Furthermore, we analysed a possible role for synchrony in development of different floret primordia as a possible factor contributing to genotypic differences in floret mortality.

## Materials and methods

### General description, treatments, and design

Details of these experiments are available in the companion paper (Ochagavía *et al.*, 2018). To recap briefly, two field experiments under potential growing conditions were carried out during 2012/13 and 2013/14 at Bell-lloc d’Urgell, NE Spain (41.63°N, 0.78°E).

**Fig. 1.**
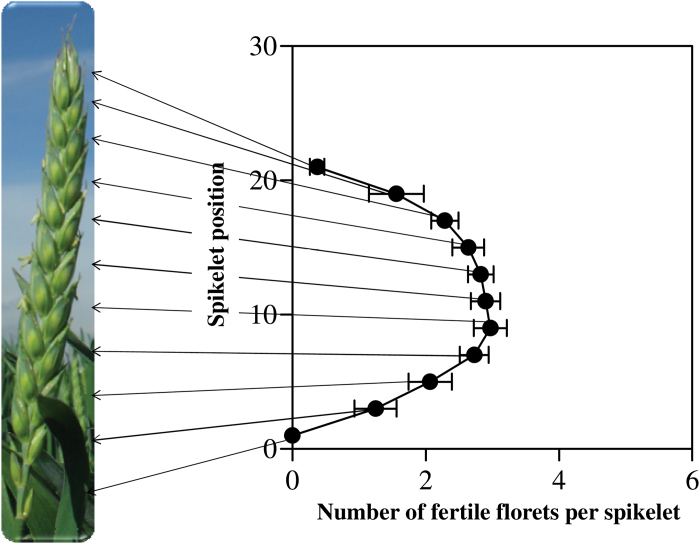
Illustration of mapping fertile florets. The number of fertile florets in each spikelet position from one side of the spike was counted from the basal to the apical positions including the terminal spikelet. (This figure is available in colour at *JXB* online.)

Treatments consisted of 13 wheat genotypes: the wild type Paragon (a spring wheat with photoperiod-sensitive alleles in all three genomes) and 12 NILs (see fig. 1 in the companion paper; Ochagavía *et al.*, 2018), five of which were single NILs (with an insensitive allele in just one of the genomes), five were double NILs (with insensitive alleles in two of the genomes), and two were triple NILs (with insensitive alleles in all three genomes). Sources of insensitivity were genotypes GS-100 (*Ppd-A1a*), Chinese Spring (*Ppd-B1a*), Sonora 64 (*Ppd-B1a* and *Ppd-D1a*), and Recital (*Ppd-B1a*). Herein we refer to these lines using a code representing the donor of the *Ppd-1* allele [the first letter(s) of the name as a subscript] in each of the genomes (A, B, and D). Naturally, when there was a sensitivity allele, the subscript was always ‘P’ (as Paragon was the only source of the sensitivity alleles) and the genome+subscript have been typed in plain text. When there were insensitivity alleles introgressed in the particular genomes of Paragon, the genomes+subscript have been typed in bold.

### Measurements and analyses

Stages of terminal spikelet and anthesis, defining the later reproductive phase when floret development takes place, were determined as explained in the companion paper (Ochagavía *et al.*, 2018). Thermal time was calculated using the mean air temperature; a base temperature of 0 ºC and the maximum temperature never above the optimum temperature for developmental progress were assumed.

The number of fertile florets (style and stigmatic branches spreading and green or yellow anthers visible) was counted in main shoot and tiller spikes from the sample taken at anthesis (0.5 m of a central row, which had exact plant density and uniformity in the sample area and its borders; see companion paper by Ochagavía *et al.*, 2018). Fertile florets were counted within each spikelet along the spike as illustrated in Fig. 1.

**Fig. 2.**
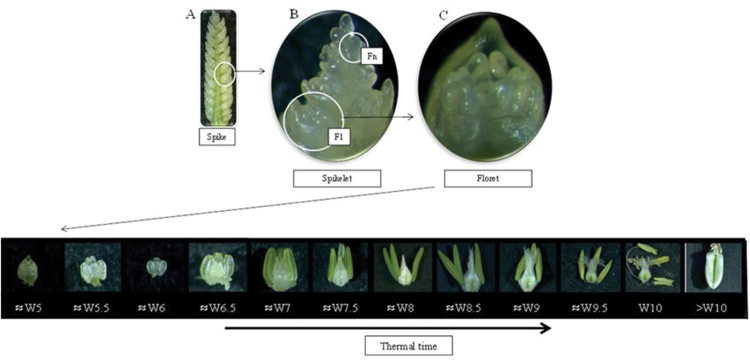
View of the entire spike with all the spikelets (A), a particular spikelet with the floret primordia (B), and a floret primordium (C) under microscopy. Changes in the pistil morphology of each floret primordium with thermal time from early stages until post-anthesis stages were followed and characterized using the scale of Waddington *et al.* (1983). (This figure is available in colour at *JXB* online.)

From the onset of stem elongation onwards, three plants from each genotype were sampled frequently (2–3 times a week, depending on the temperature). The spikes of the main shoots were dissected under a microscope (Leica MZ 7.5, Leica Microscopy System Ltd, Heerbrugg, Switzerland) and then within central spikelets floret primordia were counted and the stage of development of each primordium was determined following the scale described by Waddington *et al.* (1983) (Fig. 2). When considering individual florets, this scale starts with stage W3.5 (stamen primordia present) and finishes with florets being fertile, stage W10 (styles curved and stigmatic branches spread wide, pollen grains on well-developed stigmatic hairs). Florets were numbered from 1 to *n*, from the closest to the most distal positions with respect to the rachis, respectively, and their developmental progress was monitored individually for each floret primordium (Fig. 2). The dynamics of floret development were determined by plotting the stage of floret development (Waddington score) against thermal time from anthesis (i.e. negative values representing timing before anthesis).

The number of living floret primordia within spikelets was plotted against thermal time from anthesis for each genotype. This shows the number of primordia that were developing normally at each sampling time in the central spikelets from the beginning of floret generation (florets were considered to be a single primordium at W3.5; before that stage, the scale refers to the stage of development of the spike as a whole, not of the individual florets) until the maximum number of floret primordia is reached (floret development generation phase). In addition, the onset of floret initiation was defined when floret 1 reached the stage of W3.5. After the maximum number of primordia initiated is reached, the floret mortality or degeneration starts until the number of fertile florets is finally established around anthesis. This number of fertile florets is therefore the end result of the balance between generation and degeneration processes and so has as components the maximum number of floret primordia and the proportion of florets that survived, which was calculated as the ratio between the number of fertile florets at anthesis and the maximum number of floret primordia developed. In order to compare genotypes, living floret dynamics were plotted together, showing differences in the duration of the floret generation/degeneration phase length, in the maximum number of floret primordia developed, and in the final number of fertile florets.

The likelihood of a relatively distal floret primordium becoming a fertile floret might be related to the synchrony of initiation of different florets. To estimate the degree of synchrony of the initiation of floret primordia for each particular treatment, we determined the timing (thermal time) of the onset of floret initiation as when each primordium reached W3.5. Once the timing of W3.5 of each floret primordium was calculated, each floret position was plotted against thermal time to its stage W3.5 and fitted a linear regression whose slope was the rate of floret primordia initiation, and its reciprocal the ‘plastochron’ for floret primordia (i.e. the average thermal time interval between the initiation of two consecutive floret primordia).

To determine the significance of genotypic differences, we subjected the data to ANOVA or *t*-tests. Fishers least significant difference (LSD) was used to test when differences between particular treatments were significant (JMP^®^ Pro version 12.0 SAS Institute Inc., Cary, NC, USA). To assess the degree of relationships between variables, linear regression analyses were performed.

### Weather conditions

**Fig. 3.**
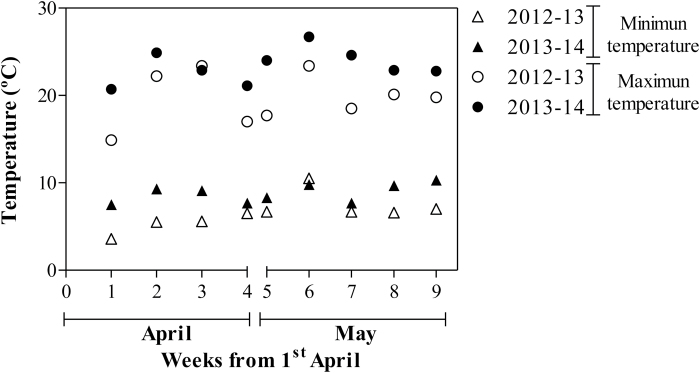
Minimum (triangles) and maximum (circles) temperatures averaged weekly for April and May in the first (open symbols) and second growing seasons (filled symbols).

All genotypes reached anthesis stage between 25 April and 21 May during the first growing season, whereas during the second they flowered between 21 April and 13 May. The main difference between growing seasons in April and May was that temperatures were higher (3.7 ºC and 4.0 ºC warmer) in the second than in the first growing season: maximum temperatures averaged for April were 18.8 ºC in the first growing season and 22.5 ºC in the second, and for May they were 20.4 ºC in the first growing season and 24.4 ºC in the second (Fig. 3).

## Results

Results (and conclusions) are based on the whole set of 13 genotypes in both experimental growing seasons. However, the number of individual relationships describing floret development dynamics was massive (floret primordia in floret positions 1–8, in 13 genotypes and two growing seasons). In order to simplify the presentation of the results, the details are explicitly shown for four genotypes (representing the ranges exhibited by the 13 lines) and for four floret positions (which represents roughly the range of floret lability). However, relationships from parameters derived from these dynamics were determined considering all genotypes. Regarding genotypes, we showed the details for the wild type (with three sensitive alleles, A_P_+B_P_+D_P_), and for one NIL with a single (A_P_+**B**_**CS**_+D_P_), one with a double (**A**_**GS**_+**B**_**CS**_+D_P_), and one with a triple (**A**_**GS**_+**B**_**CS**_+**D**_**S**_) substitution with insensitive alleles. For floret positions, the details are shown for floret primordia with either very stable final outcome (florets 1, always ending in a fertile floret; and 6, always dying before these could become a fertile floret) or very labile (florets 3 and 4, which, depending on the conditions, developed normally to become fertile or died during the developmental process). Thus, the most proximal floret (#1) represents cases in which complete development is always achieved (florets 1 and 2), the sixth floret primordium from the rachis is never developed enough to reach the fertile floret stage at anthesis (fairly representing primordia that never complete their development achieving the stage of fertile florets; florets 5/6–8 or more distal), and florets 3 and 4 are the most labile positions in which the effects of treatments affecting floret development to reach a fertile floret can be seen most noticeably.

### Number of fertile florets at anthesis

**Fig. 4.**
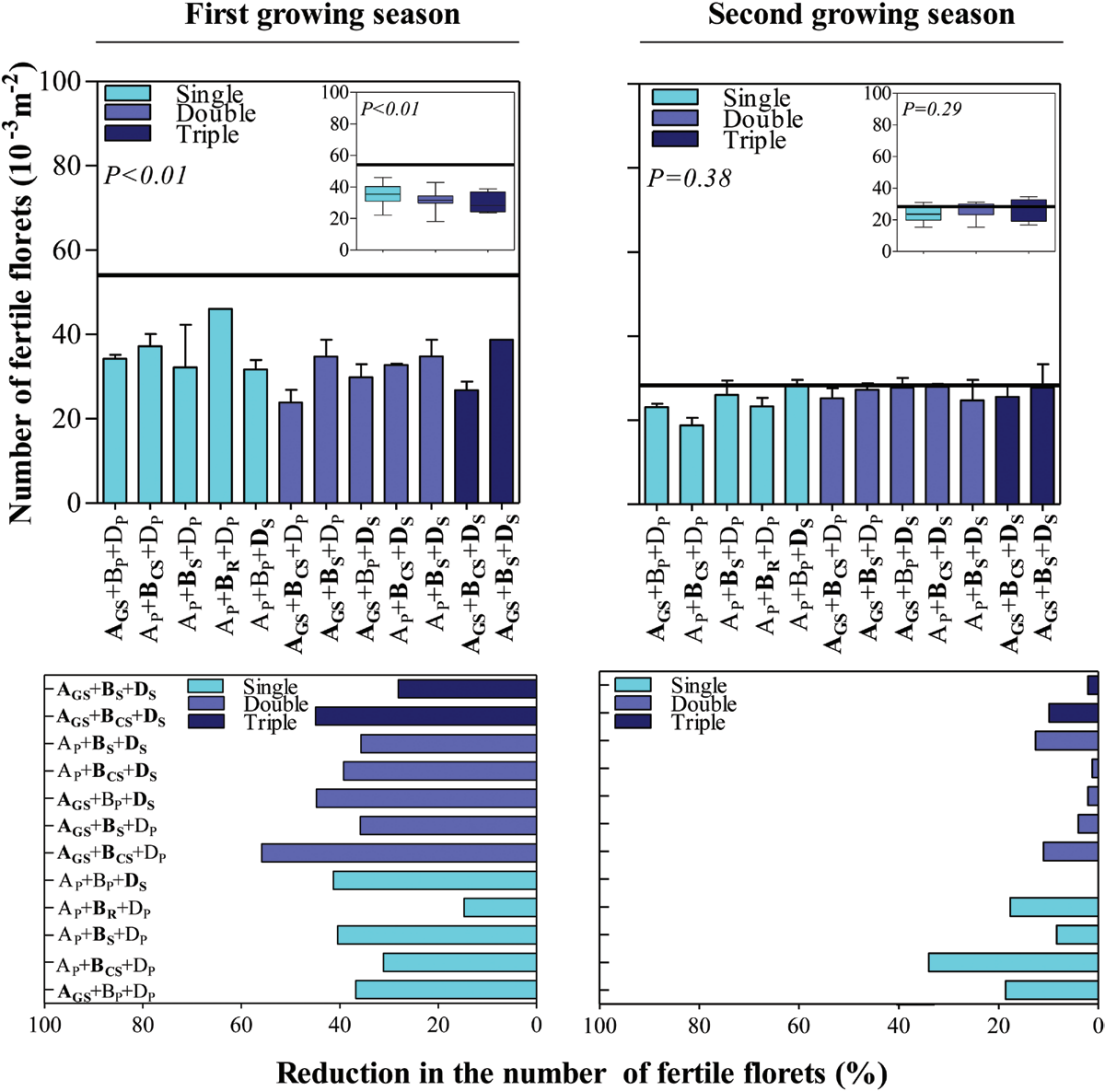
Top panels: number of fertile florets for each of the *Ppd* NILs (bars) and the wild-type Paragon (black line) during the first (left panel) and second growing seasons (right panel). Error bars stand for the SE. Insets in each panel are the box plots grouping the NILs with the single, double, or triple doses of *Ppd* alleles introgressed. Bottom panels: reduction in the number of fertile florets between each of the *Ppd* NILs and the wild-type Paragon during the first (left panel) and second growing seasons (right panel). (This figure is available in colour at *JXB* online.)

All NILs developed fewer fertile florets at anthesis than Paragon, although the differences were only consistently significant in the first growing season (Fig. 4, top left panel) while only a consistent trend, though mostly non-significant, was evident in the second growing season (Fig. 4, top right panel). In the first season, the reductions ranged from <20% to >40% among the NILs with a single dose of insensitivity, and between almost 30% and 60% when two or three insensitivity alleles were introgressed (Fig. 4, bottom left panel). In the second season, all NILs also exhibited fewer fertile florets at anthesis than Paragon, but only in three of the five single NILs (**A**_**GS**_+B_P_+D_P_, A_P_+**B**_**CS**_+D_P_, and A_P_+**B**_**R**_+D_P_) was the reduction noticeable, and only in the second was it also statistically significant with respect to Paragon (Fig. 4, top right panel).

Comparing the NILs with only one insensitivity allele, the strongest reduction in final number of fertile florets was observed with the insensitivity introgressed in the D genome in the first growing season (Fig. 4, bottom left panel), but this homoeoallele was not consistently the strongest in the second season (Fig. 4, bottom right panel) and the effect of one of the NILs carrying *Ppd-B1a* from Sonora 64 was equally strong as the insensitivity associated with *Ppd-D1a* (Fig. 4, left panels). Analysing the data of the first growing season in which the introgression of *Ppd-1a* alleles had a consistent significant effect, there was a clear overall inverse relationship between the number of insensitivity alleles and number of fertile florets (Fig. 4, top left panel, inset); but that was true on average, while some individual NILs with a single insensitivity allele had stronger reductions in number of fertile florets than some particular NILs with two or three *Ppd-1a* introgressed (Fig. 4, left panels). Even when considering the average of all NILs with a particular dose of *Ppd-1a* alleles, the increased reduction in number of fertile florets with increased doses of insensitivity alleles revealed that the effect was not additive.

### Mapping fertile florets

**Fig. 5.**
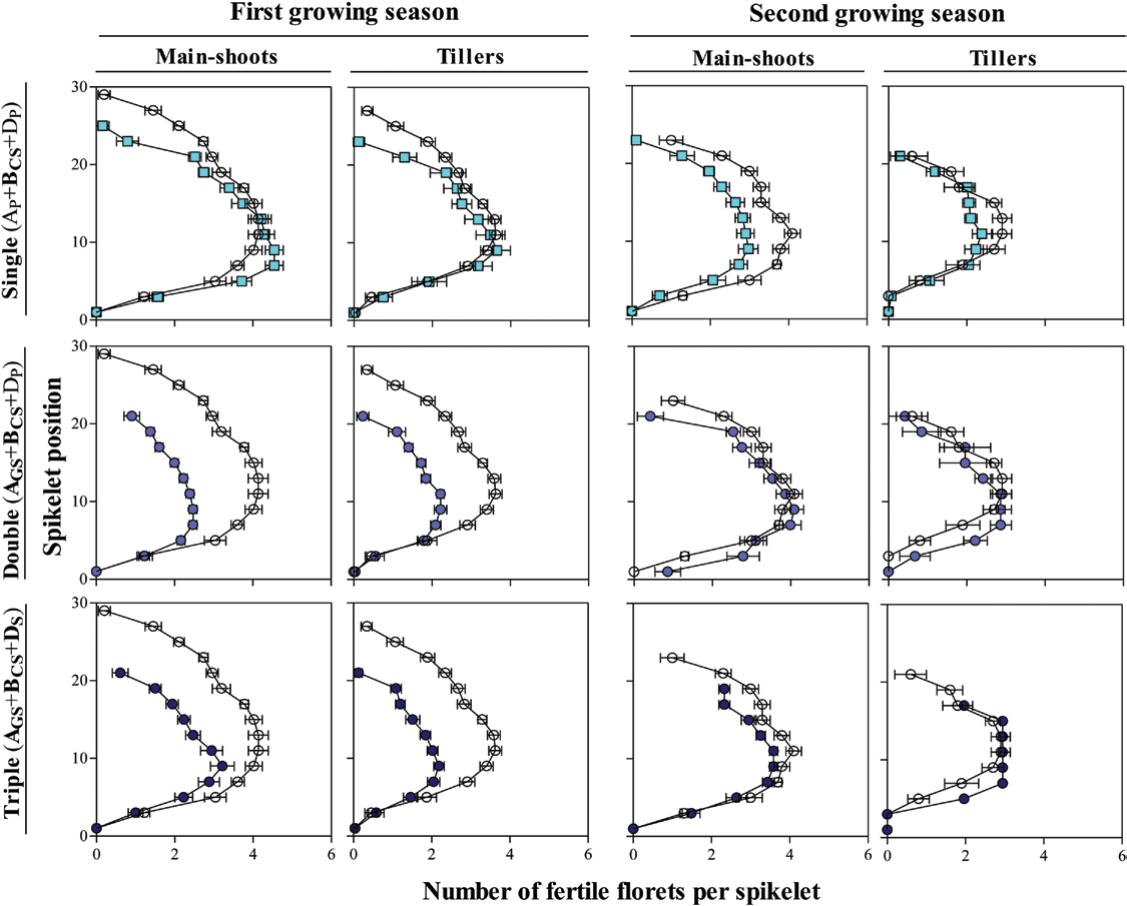
Mapping of fertile florets (fertility of each spikelet position on the main shoot or tiller spikes) for the selected NILs with one (A_P_+**B**_**CS**_+D_P_, top panels), two (**A**_**GS**_+**B**_**CS**_+D_P_, middle panels), or three (**A**_**GS**_+**B**_**CS**_+**D**_**S**_, bottom panels) insensitivity alleles in comparison with Paragon (A_P_+B_P_+D_P_, open circles) during the first (two left columns of panels) and second growing seasons (two right columns of panels). Each data point is the average of all replicates, and within each replicate the value was the average of four plants and the segment in each data point stands for the SE (not visible when smaller than the size of the symbol or in the absence of replicates in **A**_**GS**_+**B**_**CS**_+**D**_**S**_ tillers). (This figure is available in colour at *JXB* online.)

**Fig. 6.**
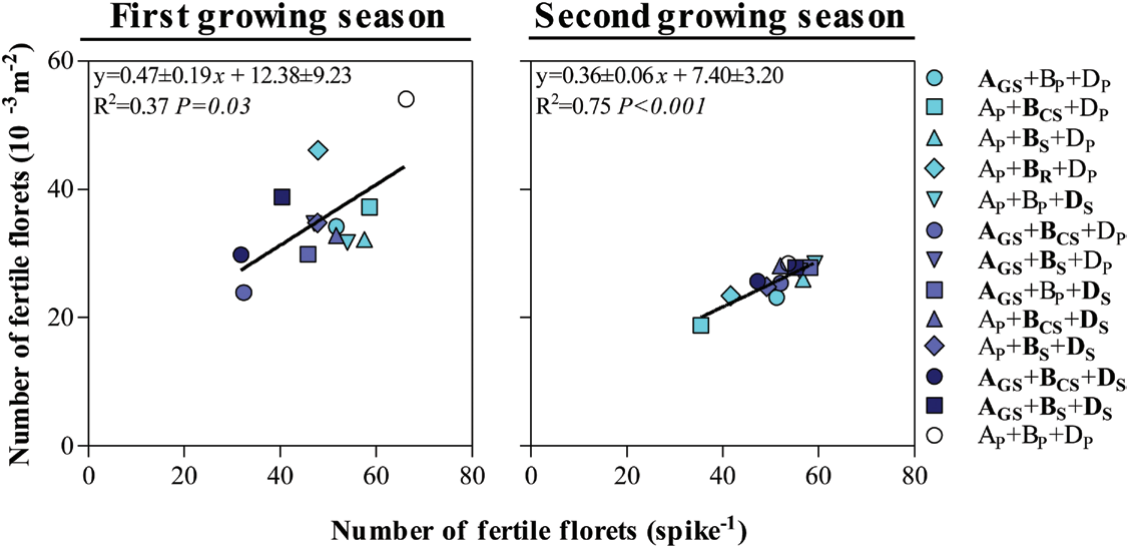
Relationship between the number of fertile florets at anthesis per square metre and the number of fertile florets per spike among the *Ppd* NILs and Paragon during the first (left column) and second growing seasons (right column). The coefficient of determination (*R*^2^) and the level of significance (*P*-value) for linear regression are shown. (This figure is available in colour at *JXB* online.)

In general, differences in the number of fertile florets along the spikes between the NILs and Paragon were extremely clear only in the first growing season (Fig. 5, left columns of panels). This is in line with the highest reduction found in the total number of fertile florets at anthesis in this growing season (Fig. 4, left panels).Comparison with Paragon carrying a single insensitivity allele showed a more noticeably reduced number of fertile florets in the apical spikelets of the main shoot and the tiller spikes in the first growing season (Fig. 5, top left panel), but not in the second growing season, when the reduction was observed across most spikelets mainly in the main shoot spikes (Fig. 5, top right panels). The NILs carrying double or triple doses of insensitivity alleles showed a marked reduction in the number of fertile florets, making the difference compared with Paragon clear in almost all spikelets of both main shoot and tiller spikes during the first growing season (Fig. 5, middle and bottom left panels), while differences in fertile florets per spike tended to be negligible in the second growing season (Fig. 5, middle and bottom right panels). Moreover, the spikes of the NILs carrying insensitivity alleles tended to be shorter than those of Paragon, again particularly clearly during the first growing season. As the differences between NILs and Paragon in fertile florets per spike resembled reasonably well those in the number of fertile florets per unit land area (Fig. 6), the main effects of *Ppd* alleles were on the fertility of the spikes rather than on the fertility of tillers. In particular, differences already explained in the number of fertile florets along the spikes in the NIL carrying one single insensitivity alleles may explain at least part of the effect on the reduction of the number of fertile florets at anthesis of ~41% and 34% in comparison with Paragon in the first and second growing season, respectively (Fig. 4, bottom left and right panels), and in the case of the NILs carrying double and triple insensitivity alleles may explain at least part of the highest differences of ~56% and ~45%, respectively, during the first growing season (Fig. 4, bottom left panel).

**Fig. 7.**
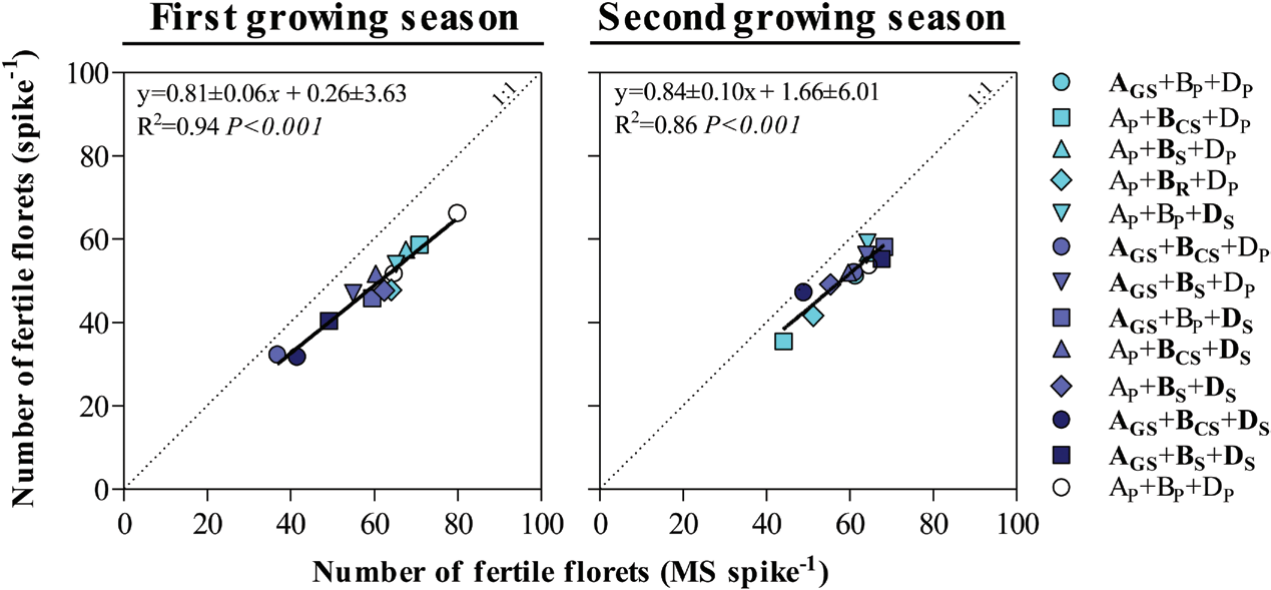
Relationship between the number of fertile florets per spike and the number of fertile florets per main shoot spike and among the *Ppd* NILs and Paragon during the first (left column) and second growing seasons (right column). The coefficient of determination (*R*^2^) and the level of significance (*P*-value) for linear regression are shown. (This figure is available in colour at *JXB* online.)

**Fig. 8.**
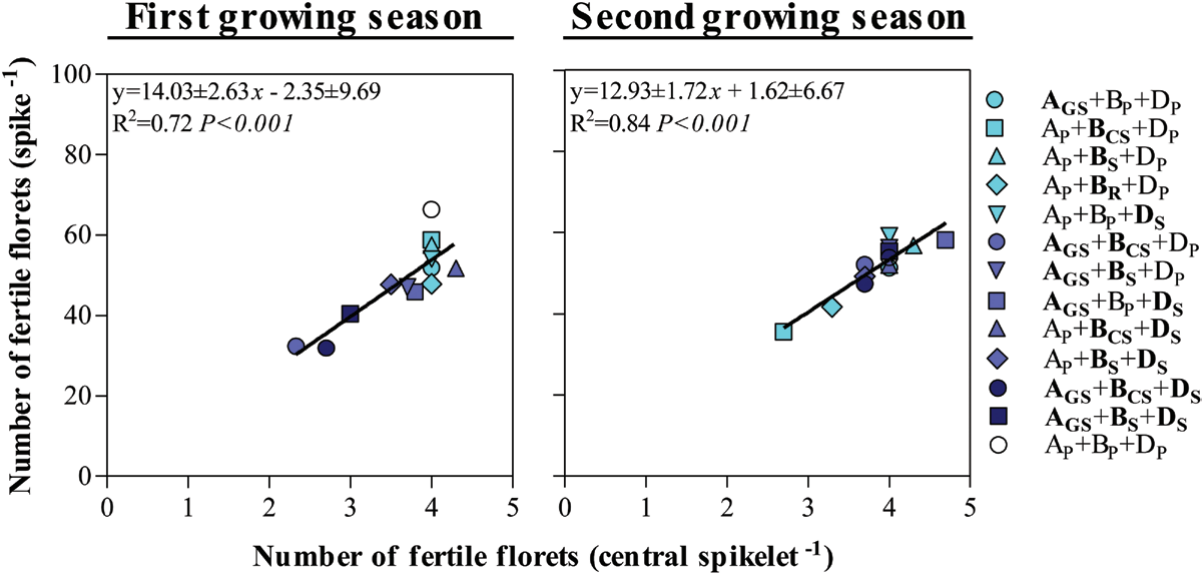
Relationship between the number of fertile florets per spike and the number of fertile florets per central spikelet among the *Ppd* NILs and Paragon during the first (left column) and second growing seasons (right column). The coefficient of determination (*R*^2^) and the level of significance (*P*-value) for linear regression are shown. (This figure is available in colour at *JXB* online.)

In this context, even though not perfect, the fertility of the main shoot spikes reflected well that of the pool of all spikes in the canopy across all NILs (Fig. 7). Naturally, the total number of fertile florets was consistently larger in the main shoot spikes than in the average of all spikes (in all lines, in both growing seasons), but most (>86%; *P*<0.001) of the differences between lines in spike fertility for the average spike in the canopy were explained by differences in fertility of the main shoot spikes. Likewise, differences in fertility seen by pooling all the spikelets in the spike were highly related to the differences in fertility of the central spikelet (Fig. 8), in which we can trace back the developmental process that lies at the origin of differences in floret fertility for each NIL.

### Floret development and living floret primordia dynamics in the central spikelets

**Fig. 9.**
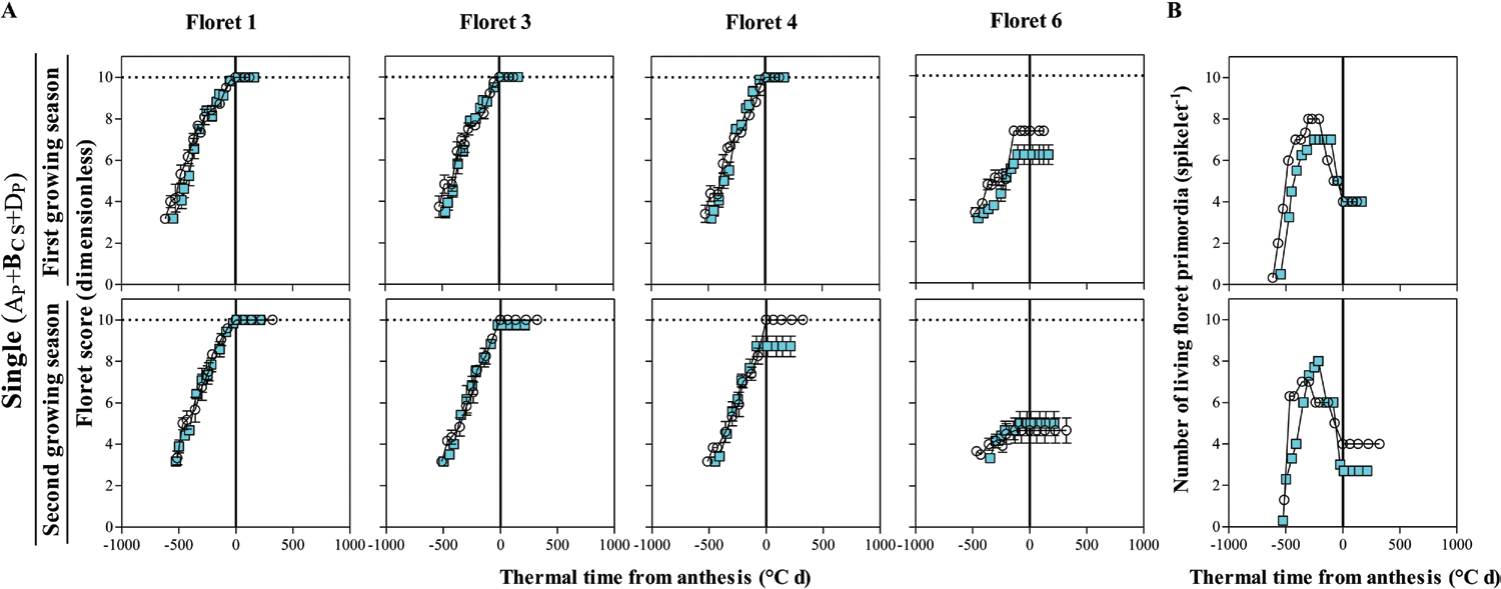
Dynamics of the floret development of F1, F3, F4, and F6 in central spikelets of the main shoot (A) and the number of living floret primordia (B) through thermal time from anthesis in A_P_+**B**_**CS**_+D_P_ carrying one single change (filled squares), in comparison with Paragon (A_P_+B_P_+D_P_, open circles) during the first (top panels) and second growing seasons (bottom panels). Data points are the average from replicates; bars represent the SE. (This figure is available in colour at *JXB* online.)

No relevant differences were found in the dynamics of developmental progress of florets 1, 3, and 4 between the NILs carrying a single insensitivity allele and Paragon during the first growing season (Fig. 9A, top panels). Although, floret 6 did not reach the fertile floret stage in any of these two genotypes, this floret primordium was clearly more developed in Paragon than in the NIL with a single insensitive allele (Fig. 9A, top panels). As the main difference in developmental rates of floret primordia in the first growing season was in floret positions which were not fertile in any case, the dynamics of floret initiation and degeneration showed a similar final number of fertile florets in the NIL and in Paragon, even though the period of floret degeneration (when survival or death of particular florets is determined) was slightly longer in the wild type (Fig. 9B, top panel). In the second growing season, no differences were found in the dynamics of floret 1 and 3 either, but the fourth floret developed normally until reaching the fertile floret stage in Paragon, while it did not do so in the NIL with one insensitive allele, and no differences were found in development of floret 6 (Fig. 9A, bottom panels). Consequently, carrying a single insensitivity allele lowered the number of fertile florets compared with Paragon, which again exhibited a longer period of floret degeneration (which might be responsible for the reduced rate of floret mortality, increasing the final number of fertile florets at the end of the floret development period; Fig 9B, bottom panel).

**Fig. 10.**
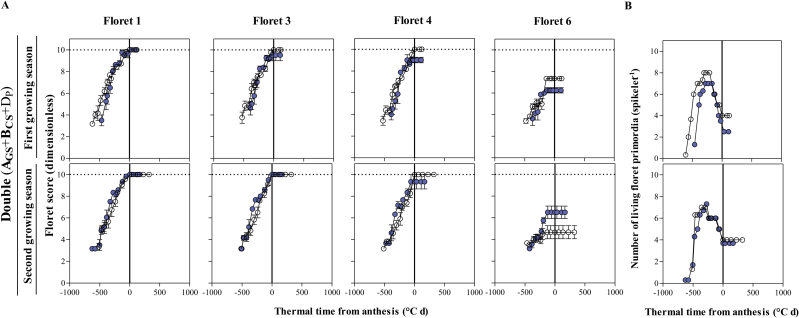
Dynamics of the floret development of F1, F3, F4, and F6 in central spikelets of the main shoot (A) and the number of living floret primordia (B) through thermal time from anthesis in **A**_**GS**_+**B**_**CS**_+D_P_ carrying double change (filled circles), in comparison with Paragon (A_P_+B_P_+D_P_, open circles) during the first (top panels) and second growing seasons (bottom panels). Data points are the average from replicates; bars represent the SE. (This figure is available in colour at *JXB* online.)

Comparing the floret development dynamics between a NIL carrying two insensitive alleles and Paragon, during the first growing season, no differences were found in floret 1 but floret 3 reached the fertile stage in all Paragon plants whereas it did so only in some of the plants of the photoperiod-insensitive NIL (Fig. 10A, top panels). In addition, floret 4 reached the fertile stage only in Paragon but never in the NIL with two insensitive alleles. Again, floret 6, which was not developed enough to reach the fertile floret stage in any genotype, presented in the first growing season a less advanced developmental stage in the NIL with two insensitive alleles than in Paragon (Fig. 10A, top panels). During the second growing season, no difference was found for florets 1 and 3, but floret 4 was always fertile in Paragon while it was only fertile in some plants of the NIL with two insensitive alleles (Fig. 10A, bottom panels). In this second season, floret 6 was actually more developed in the NIL with two insensitive alleles than in the wild type (Fig. 10A, bottom panels). Regarding the dynamics of the number of living floret primordia, in both growing seasons the NIL with two insensitive alleles had a shorter period of floret death and a lower number of fertile florets at anthesis; in both cases, the differences were clearer in the first than in the second growing season (Fig. 10B).

**Fig. 11.**
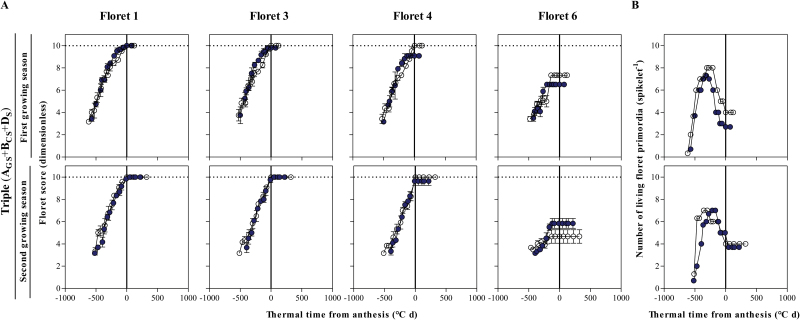
Dynamics of the floret development of F1, F3, F4, and F6 in central spikelets of the main shoot (A) and the number of living floret primordia (B) through thermal time from anthesis in **A**_**GS**_+**B**_**CS**_+**D**_**S**_ carrying triple change (filled circles), in comparison with Paragon (A_P_+B_P_+D_P_, open circles) during the first (top panels) and second growing seasons (bottom panels). Data points are the average from replicates; bars represent the SE. (This figure is available in colour at *JXB* online.)

In the case of the NIL carrying three insensitivity alleles in comparison with Paragon, developmental progress towards becoming fertile structures of individual florets was rather similar to the other two NILs, although more similar to the NIL with two than to that with one insensitive allele (Fig. 11A). Regarding the resulting dynamics of generation and degeneration of floret primordia, the NIL with the three insensitive alleles presented a reduction in the number of fertile florets at the end of the process, which was clearer in the first than in the second growing season, but the reduction in the duration of the floret death period was clear only in the second season (Fig. 11B).

Similar differences from the wild type for the overall patterns of progress of floret development for individual floret positions and of dynamics of living floret primordia were observed in most of the other NILs also with similar differences in magnitude of responses between growing seasons (data not shown), although there were some exceptions in which the NILs with insensitivity finally produced more fertile florets than Paragon in the central spikelets (e.g. A_P_+**B**_**CS**_+**D**_**S**_ in the first growing season; A_P_+**B**_**S**_+D_P_ and **A**_**GS**_+B_P_+**D**_**S**_ in the second growing season; data not shown). However, these exceptions have to be considered carefully as they are more apparent than real: the insensitivity alleles also induced production of fewer spikelets per spike and consequently the apparently higher fertility of these exceptional cases mostly reflects this difference in spike structure, and in all cases introgression of insensitivity alleles reduced the overall fertility of the spikes.

**Fig. 12.**
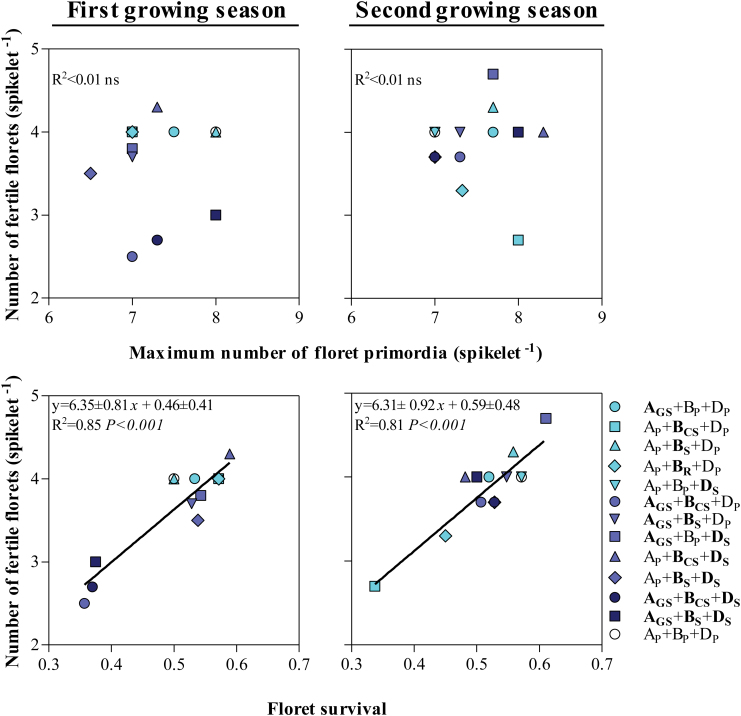
Relationship between the number of fertile florets at anthesis and the maximum number of floret primordia developed in the central spikelets of the main shoot spikes (top panels), and relationship between the number of fertile florets at anthesis and floret survival in the central spikelets from the main shoots spikes (bottom panels) among *Ppd* NILs and Paragon during the first (left panels) and second growing seasons (right panels). The coefficient of determination (*R*^2^) and the level of significance (*P*-value) for linear regression are shown. (This figure is available in colour at *JXB* online.)

In general, the effects of the photoperiod insensitivity alleles on the final number of fertile florets were mainly due to their effects on floret death determining the level of floret survival. Although there was variation in the maximum number of floret primordia initiated, over all NILs there was no relationship between the number of fertile florets at anthesis and the maximum number of floret primordia developed in any of the two growing seasons (Fig. 12, top panels). On the other hand, the number of fertile florets was highly and positively related to floret survival in both growing seasons (Fig 12, bottom panels).

At least in part, the difference in likelihood of a floret primordium becoming a fertile floret may be related to the differential timing between the onset of development of floret primordia at distal and proximal positions (i.e. the time elapsed between the initiation of different florets might be responsible for the rate of floret death, and the synchrony in the initiation of florets at different positions would play a role in determining the rate of floret survival.

### Synchrony in floret primordia initiation

**Fig. 13.**
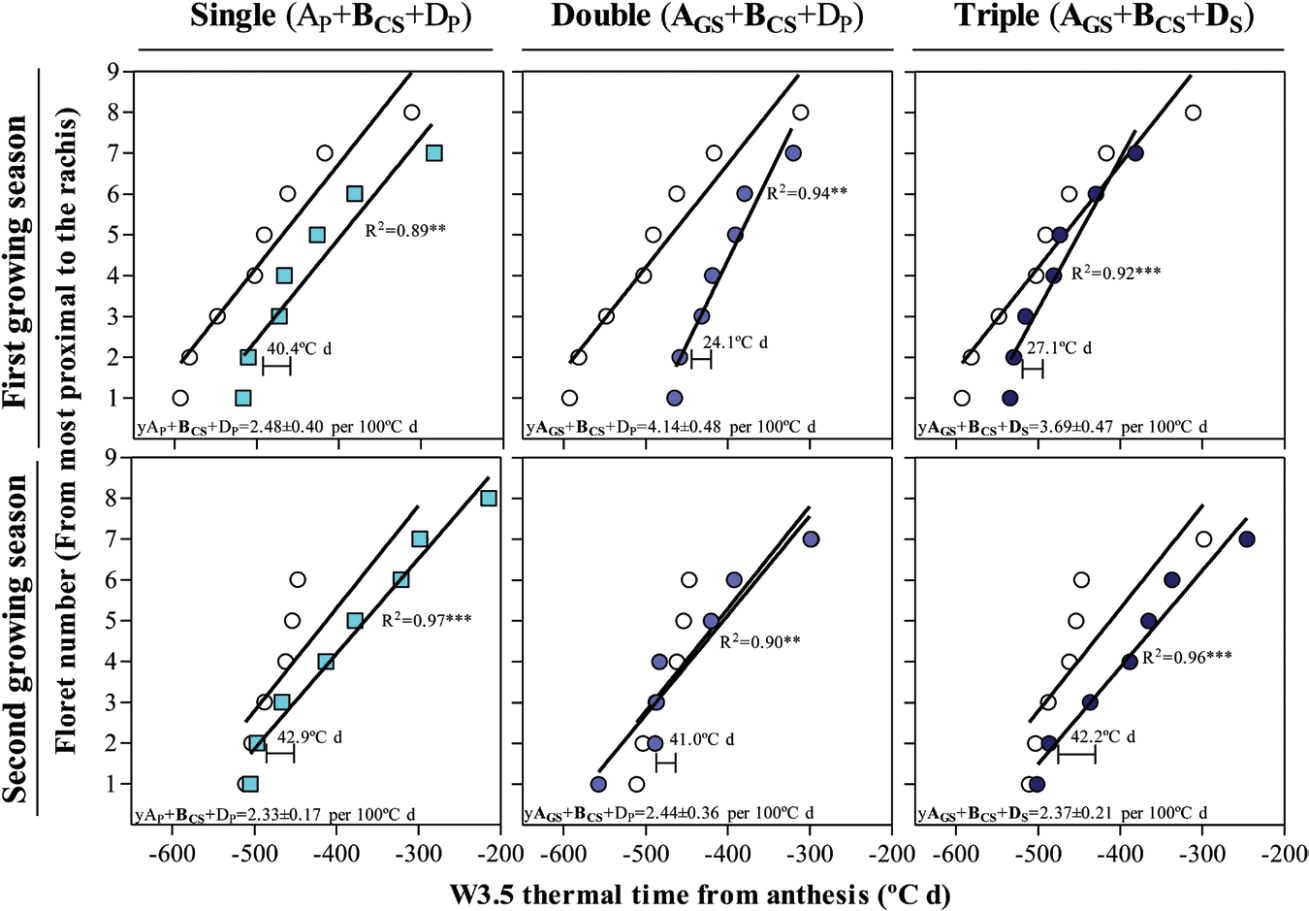
Timing of W3.5 for each floret primordium through thermal time from anthesis in selected NILs carrying a single (A_P_+**B**_**CS**_+D_P_, left column), double (**A**_**GS**_+**B**_**CS**_+D_P_, middle column), and triple change (**A**_**GS**_+**B**_**CS**_+**D**_**S**_, right column) in comparison with Paragon (A_P_+B_P_+D_P_, open circles) whose rate of floret initiation was 2.53 ± 0.30 (*R*^2^=0.92***) during the first growing season (top panels) while it was 2.52 ± 0.72 (*R*^2^=0.71*) during the second growing seasons (bottom panels). In all the cases, the floret initiation rates are expressed in florets per 100 °Cd. The coefficient of determination (*R*^2^) and the level of significance for each NIL linear regression are shown. The inset in each panel shows the thermal time between the appearance of two following floret primordia; for Paragon it was 39.5°Cd and 39.7 °Cd during the first and the second growing seasons, respectively. (This figure is available in colour at *JXB* online.)

The relationships between the floret position and the thermal time before anthesis when each floret was initiated (reached the W3.5 stage) were curvilinear in almost all cases, mainly because the most distal primordium (or the two most distal ones) tended to be noticeably delayed with respect to the rate of initiation exhibited by the previous primordia (remarkably so in the wild type in the second growing season) (Fig. 13). Disregarding this curvilinearity, as the trend has a strong linear component, in all cases the linear regression showed a highly significant coefficient of determination (*R*^2^ ranging from 0.71, *P*<0.05, to 0.98, *P*<0.001; Fig. 13; Supplementary Figs S1, S2 at *JXB* online). During the first growing season, there was a trend for an increased rate of floret primordia initiation with the introgression of photoperiod insensitivity alleles; the difference from the rate of the wild type was not significant for the NIL carrying a single dose of insensitivity alleles (Fig. 13, top left panel) but it was larger and significant for the NILs carrying double and triple doses of insensitivity alleles (Fig. 13, top middle and top right panels). During the second growing season, differences were not significant (Fig. 13, bottom panels). These results reflected well the overall results observed for each of the NILs (Supplementary Figs S1, S2).

However, the differences in rates of floret initiation and consequently in synchrony between the initiation of early- and late-initiated floret primordia, which were clear in the first year and only minor in the second, seemed largely inconsequential for the maximum number of floret primordia initiated, the floret survival rate, and the number of fertile florets at anthesis (*R*^2^ ranging from 0.01^ns^ to 0.12^ns^).

Finally, there was not a clear relationship between (i) the maximum number of floret primordia initiated; (ii) the floret survival rate; or (iii) the resulting number of fertile florets and the stage of development of the floret primordium nearest the rachis (the most advanced floret) at the time when the maximum number of floret primordia was reached (the onset of floret death) (*R*^2^ ranging from <0.01^ns^ to 0.21^ns^).

### Duration of floret development

**Fig. 14.**
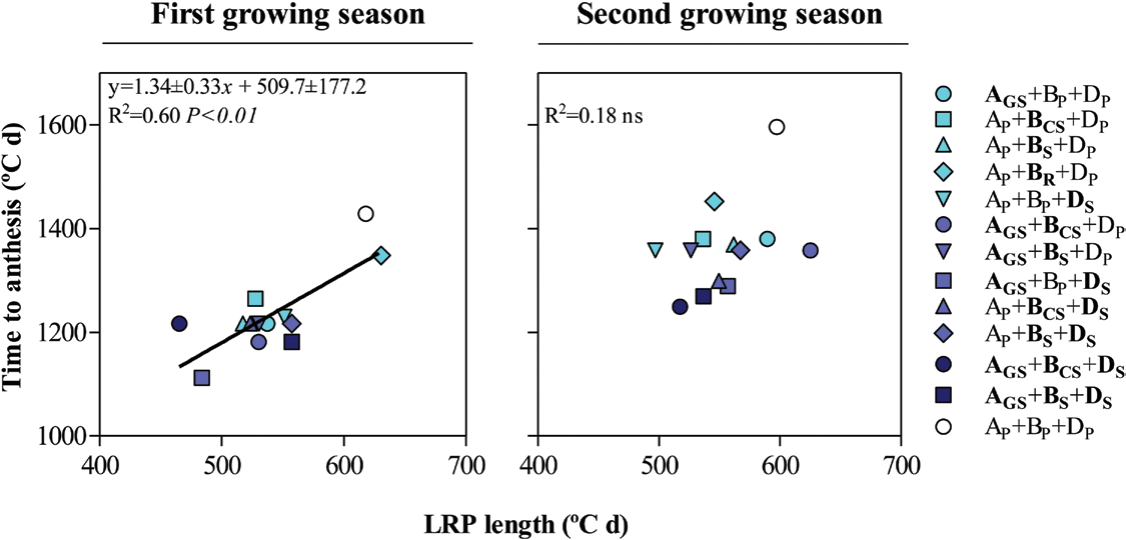
Relationship between time to anthesis and the late reproductive phase length (LRP) among the NILs and Paragon during the first (left) and the second growing seasons (right). The coefficient of determination (*R*^2^) and the level of significance (*P*-value) for linear regression are shown. (This figure is available in colour at *JXB* online.)

Carrying the insensitivity allele(s) in most cases shortened time to anthesis due to a reduction in the duration of the late reproductive phase from terminal spikelet initiation to anthesis. This result was clear and highly significant in the first growing season, but only showed a trend in the second growing season (Fig. 14).

With similarity to what happened with the synchrony in floret development, there was no clear relationship between the floret survival or its consequence, the number of fertile florets, and any of the durations of floret development. Neither the whole period of stem elongation, the specific period of floret initiation, the period of floret primordia generation, nor the period of floret degeneration was relevant for explaining by themselves the rate of floret mortality (*R*^2^ ranging from <0.01^ns^ to 0.08^ns^) or the final number of fertile florets (*R*^2^ ranging from <0.01^ns^ to 0.15^ns^).

## Discussion

Carrying photoperiod insensitivity alleles caused a reduction in the number of fertile florets, which is in agreement with the general effects of *Ppd* alleles on the number of organs being developed during the phase whose duration is affected by these genes. This sort of parallelism has been established for leaf and spikelet primordia (i.e. when photoperiod changes the duration of a phase it changes more or less proportionally the number of primordia initiated; Slafer and Rawson, 1994; Ochagavía *et al.*, 2018), but to the best of our knowledge the same effect on fertile florets was hypothesized (Slafer *et al.*, 2001) but not demonstrated, and, as far as we are aware, this is the first time they are documented for floret primordia. It might not have been similar because, unlike the determination of leaves and spikelets in which all the primordia initiated contribute to the final number achieved, the number of fertile florets at anthesis is the outcome of a process of generation followed by degeneration of floret primordia. In fact, the processes are not parallel: in leaf and to some degree in spikelet, in the initiation phase it seems that *Ppd* alleles alter the rate of phasic development (changing the duration of the phase; González *et al.*, 2002; Whitechurch and Slafer, 2001, 2002; Foulkes *et al.*, 2004) but do not noticeably affect the rate of leaf initiation and, consequently, the number of organs initiated is rather linearly related to the duration of the phase when they are initiated (Slafer and Rawson, 1994). In this study, it was shown that synchrony of floret initiation was improved with the introgression of insensitivity alleles, which implies that unlike the development of leaf primordia (and to a certain degree spikelet primordia; Ochagavía *et al.*, 2018), the rate of floret primordia initiation was positively affected by insensitivity to photoperiod, to the same extent as these alleles positively affect the rate of phasic development of the late reproductive phase. Consequently, the maximum number of floret primordia was largely unaffected by *Ppd* alleles. Thus, the effect on the number of fertile florets seemed to have occurred indirectly, through the reduction in time, making fewer resources available for the most labile floret primordia to continue developing normally towards the production of fertile florets during the floret mortality phase. This would be in line with much of the available evidence showing that the fate of floret development seems to depend upon the allocation of resources (e.g. González *et al.*, 2005*a*; Ferrante *et al.*, 2010; Guo and Schnurbusch, 2015). Thus, despite the observation that insensitivity alleles frequently improved the synchrony in floret primordia initiation, there was compensation with the effects of these alleles in shortening the duration of the processes, and synchrony was not related to floret survival or final number of fertile florets.

The effect of *Ppd* insensitivity alleles on the number of spike-bearing tillers was negligible due to trade-offs between tillering and tiller mortality (Ochagavía *et al.*, 2017), and differences in the number of fertile florets per square metre observed among the NILs and Paragon were therefore almost exclusively due to the effects of these alleles on spike fertility. This is somehow expected as the likelihood of the alleles reducing the number of spikes per unit land area is lower than that of reducing the number of florets per spike, as the latter implies a more refined regulation (Slafer *et al.*, 2014), and thus more consistent with the strength of the effects of the alleles on the reproductive phases. That is why differences in the number of fertile florets per square metre were well explained by differences in the number of fertile florets seen in the spikes.

Also in line with previous reports (González *et al.*, 2005*a*; González-Navarro *et al.*, 2015; Guo *et al.*, 2016, 2017), differences seen in the number of fertile florets in the spikelets were better related to floret survival than to the maximum number of primordia developed, and seemed not to be related to particular developmental stages of the more advanced florets.

The strength of the effects on the traits measured was rather independent of the particular genome of Paragon in which the sensitive allele was substituted by an insensitive allele. This is in line with differences reported in the order of strength of the genomes for time to heading or anthesis; while Scarth and Law (1984); Law (1987); Worland (1996); Stelmakh (1998); Worland *et al.* (1998); González *et al.* (2005*c*), and Díaz *et al.* (2012) reported the *Ppd-D1* gene to be the strongest, Tanio and Kato (2007) remarked on the importance of *Ppd-B1* comparable with *Ppd-D1*, and Bentley *et al.* (2011) reported the strength in the A genome in synthetic hexaploid wheats with intermediate effects between D and B. This emphasizes the importance of the allelic form (source) being used for substitution in a particular genome. In addition, increasing the dose of insensitivity alleles tended to produce larger differences in traits, although it was not fully consistent and in any case the effect was not fully additive. Lack of consistency may well again be due to the effect of the source of the alleles, either due to allelic differences in the *Ppd-1* gene, linked alleles, or even unlinked background effects contributed by the donor cultivar used for backcrossing. These results and interpretations are in line not only with those of other traits considered in the same study (Ochagavía *et al.*, 2017, 2018), but also with a previous discussion in González *et al.* (2005*c*) who compared the effects of *Ppd-D1* and *Ppd-B1*. That discussion highlighted lack of consistency, implying that other elements would be more relevant than the genome in which the allele was substituted in determining the strength of the effect, remarking on the importance of the source of the alleles over that of the genome in which the insensitive allele substituted the sensitive allele, something already hypothesized a long time ago (Scarth and Law, 1984).

As explained in the Results, the effects of introgressing the *Ppd* alleles were evident in the first growing season, while in the second season they became only trends. Attempting to understand why the responses were different in the two seasons, we analysed different climatic characteristics (soils were similar, and in both seasons experiments were well irrigated and fertilized). The hypothesis was that perhaps an interaction with temperature could be possible (see also Ochagavía *et al.*, 2017). This was based on the fact that temperature and photoperiod seem to act interactively, rather than additively (Slafer and Rawson, 1996). In fact, there was an increment in maximum temperatures at the field around the flowering time in the second growing season with respect to the first one. Floral development has been identified as sensitive to climatic stress including pollen formation (Saini and Aspinall, 1982; Lalonde *et al.*, 1997), and floral abnormalities induced by heat stress (i.e. stamen hypoplasia and pistil hyperplasia) were also reported in rice (Takeoka *et al.*, 1991). A modelling exercise parameterized by flowering observations indicated that the temporal and spatial variability of anther activity within and between spikes may influence the relative resilience of wheat to climatic events (Lukac *et al.*, 2012). Furthermore, even though the lines with different degrees of insensitivity and also Paragon presented a lower number of fertile florets during the second growing season, the effect seemed to be stronger in Paragon rather than in the NILs (that is why significances of the differences were mostly lost in the second growing season), suggesting an interaction between temperatures and photoperiod sensitivity for floret development that seems worthy of further consideration.

In conclusion, photoperiod insensitivity did affect spike fertility. Carrying photoperiod insensitivity reduced the number of fertile florets due to a combination of effects on floret development phase length, the rate of floret appearance, and floret survival. However, in quantitative terms, as *Ppd-1a* alleles also accelerated the rate of floret initiation (comparing different floret positions) improving the synchrony of floret development, as well as the rate of floret development (for each particular floret position), the reduction in number of fertile florets, even in the growing condition where the reduction was largest, was less than proportional to the reduction in duration of the phase. Therefore, adjusting phenology to improve adaptation exploiting the introgression of *Ppd-1a* alleles seems to drive only relatively minor trade-offs in spike fertility.

## Supplementary data

Supplementary data are available at *JXB* online.

Fig. S1. Timing of W3.5 for each floret primordium through thermal time from anthesis in NILs in comparison with Paragon during the first growing season.

Fig. S2. Timing of W3.5 for each floret primordium through thermal time from anthesis in NILs in comparison with Paragon during the second growing season.

Supplementary Figure S1-S2
