# Supplementary material for: Dynamics of floret initiation/death determining spike fertility in wheat as affected by Ppd genes under field conditions
Source: J Exp Bot. 2018 Mar 19;69(10):2633–45. doi: 10.1093/jxb/ery105 (PMC5920323; doi:10.1093/jxb/ery105)
Supplement: Supplementary Figure S1-S2 [file ery105_suppl_supplementary_figure_s1-s2.pdf]

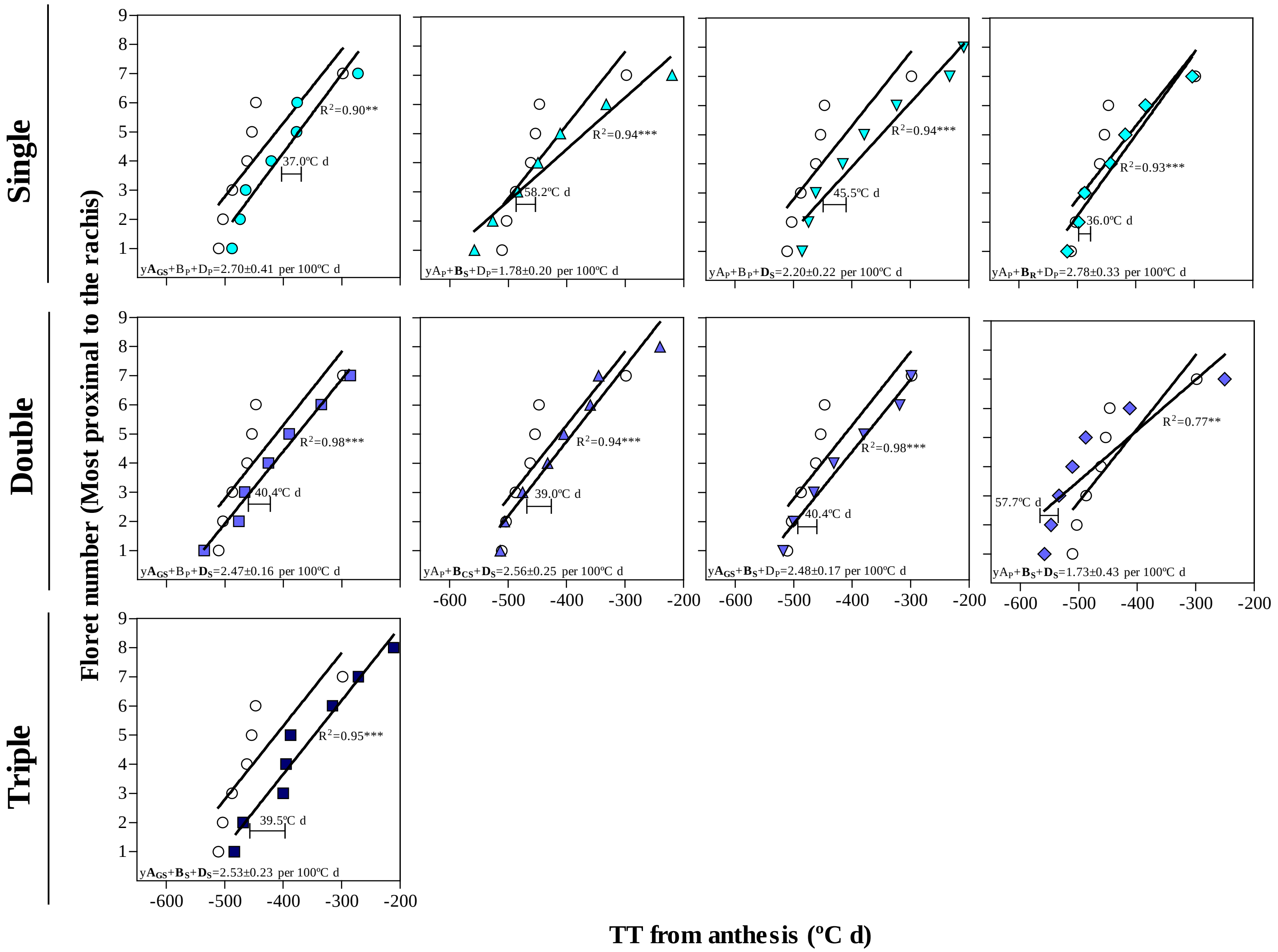

Fig. S1. Timing of W3.5 for each floret primordium through thermal time from anthesis in NILs carrying one single (light blue symbols, *top panels*), double (blue symbols, *middle panels*) and triple change (dark blue symbols, *bottom panels*) in comparison to Paragon (A_P+B_P+D_P, open circles) which rate of floret initiation was: 2.53±0.30 (R^2=0.92***), during the first growing season. In all the cases the floret initiation rates are expressed in florets per 100°C d. The coefficient of determination (R^2) and the level of significance for each NIL linear regression are shown. Inset each panel, thermal time between the appearance of two following floret primordia are indicated, for Paragon was 39.5ºC d.

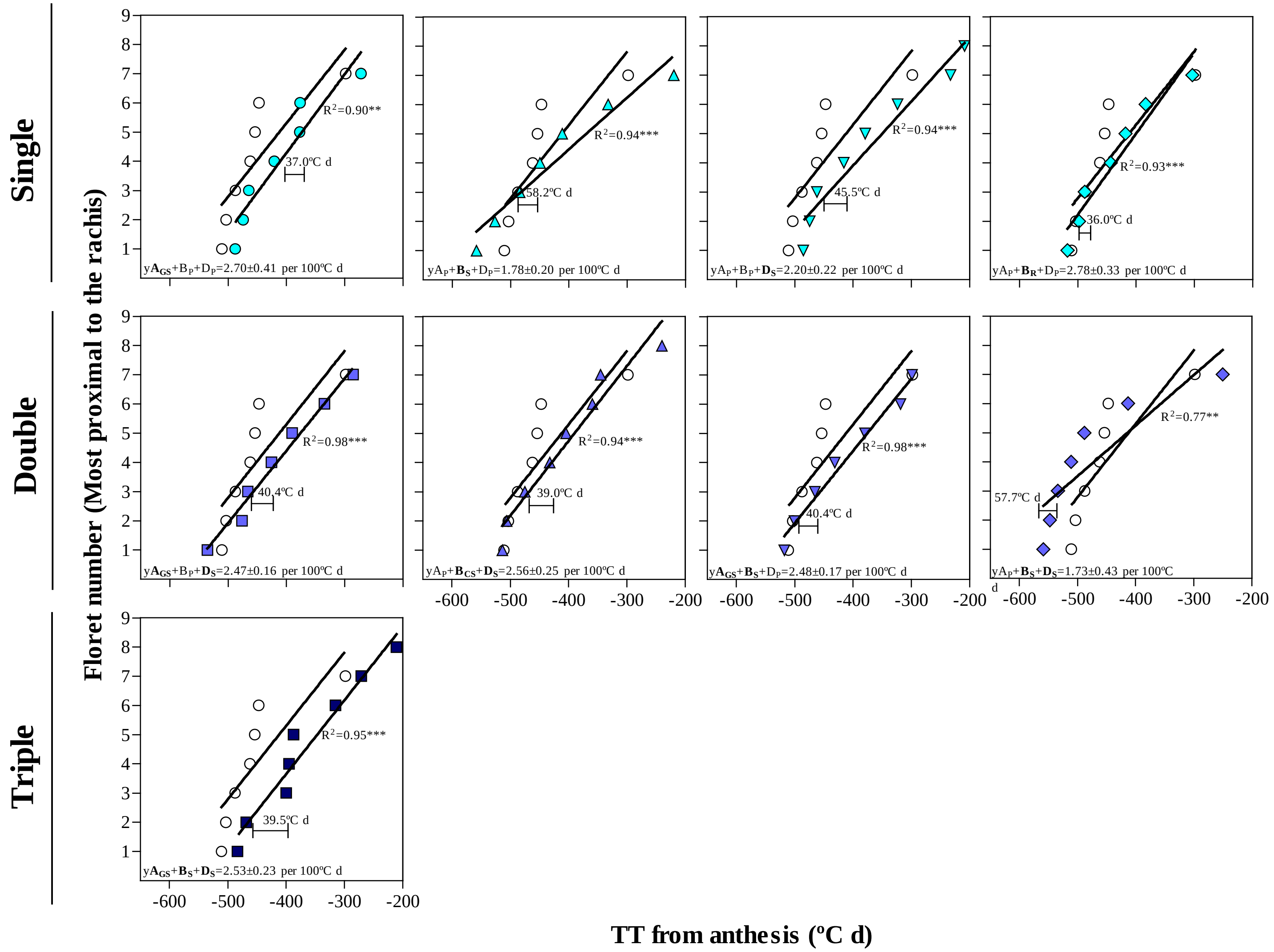

Fig. S2. Timing of W3.5 for each floret primordium through thermal time from anthesis in NILs carrying one single (light blue symbols, *top panels*), double (blue symbols, *middle panels*) and triple change (dark blue symbols, *bottom panels*) in comparison to Paragon (A_P+B_P+D_P, open circles) which rate of floret initiation was: 2.52±0.72 (R^2=0.71*), during the second growing season. In all the cases the floret initiation rates are expressed in florets per 100°C d. The coefficient of determination (R^2) and the level of significance for each NIL linear regression are shown. Inset each panel, thermal time between the appearance of two following floret primordia are indicated, for Paragon was 39.7°C d.
